# Influence of cavity design preparation on stress values in maxillary premolar: a finite element analysis

**DOI:** 10.3325/cmj.2012.53.568

**Published:** 2012-12

**Authors:** Ivana Kantardžić, Darko Vasiljević, Larisa Blažić, Ognjan Lužanin

**Affiliations:** 1 University of Novi Sad, Faculty of Medicine, School of Dentistry, Novi Sad, Serbia; 2 University of Belgrade, Institute of Physics, Belgrade, Serbia; 3 University of Novi Sad, Faculty of Medicine, School of Dentistry, Vojvodina Clinic of Dentistry, Novi Sad, Serbia; 4 University of Novi Sad, Faculty of Technical Sciences, Department of Production Engineering, Novi Sad, Serbia

## Abstract

**Aim:**

To analyze the influence of cavity design preparation on stress values in three-dimensional (3D) solid model of maxillary premolar restored with resin composite.

**Methods:**

3D solid model of maxillary second premolar was designed using computed-tomography (CT) data. Based on a factorial experiment, 9 different mesio-occlusal-distal (MOD) cavity designs were simulated, with three cavity wall thicknesses (1.5 mm, 2.25 mm, 3.0 mm), and three cusp reduction procedures (without cusp reduction, 2.0 mm palatal cusp reduction, 2.0 mm palatal and buccal cusp reduction). All MOD cavities were simulated with direct resin composite restoration (Gradia Direct Posterior, GC, Japan). Finite element analysis (FEA) was used to calculate von Mises stress values.

**Results:**

The von Mises stresses in enamel, dentin, and resin composite were 79.3-233.6 MPa, 26.0-32.9 MPa, and 180.2-252.2 MPa, respectively. Considering the influence of cavity design parameters, cuspal reduction (92.97%) and cavity wall thickness (3.06%) significantly (*P* < 0.05) determined the magnitude of stress values in enamel. The influence of cavity design parameters on stress values in dentin and resin composite was not significant. When stresses for enamel, dentine, and resin composite were considered all together, palatal cusp coverage was revealed as an optimal option. Cavity wall thickness did not show a significant effect on stress values.

**Conclusion:**

Based on numerical simulations, a palatal cusp reduction could be suggested for revealing lower stress values in dental tissues and restorative material. This type of cavity design should contribute to better biomechanical behavior of tooth-restoration complex, consequently providing the long-lasting clinical results.

In the recent years, there has been an increasing interest in the research of biomechanical aspects of biomaterials and human tissues (1-3). Although studies conducted in vivo and in vitro have provided some of the answers in this field, dental and medical research is usually costly, and may be ethically questionable and time-consuming (4,5). Because of this, the use of numerical models and in vitro simulations became a valuable tool for saving time and money associated with laboratory and clinical research (6). Previous studies have reported different techniques for generating three-dimensional (3D) solid models of the teeth (7-9). Nowadays, technological development brings new possibilities for efficient generation of sophisticated 3D solid models. For example, using specialized software, these models can be generated based on computed-tomography (CT) scan data (5,10-12). In addition, the application of finite element analysis (FEA) allows calculation of stress and strain within tooth structure and biomaterials, which can hardly be measured in vivo (13).

Cavity design preparation has a great impact on stress values and fracture resistance of a tooth (7,14-16). It is a factor of a paramount importance, especially in cases of restoring maxillary premolars with extensive mesio-occlusal-distal (MOD) cavities (14,17,18). From the biomechanical point of view, different opinions have been reported on the most appropriate restorative procedure in such cases. Kuijs et al have found that ceramic, indirect resin composite and direct resin composite provide comparable fatigue resistance in a cusp replacing restorations (19). These findings were supported by clinical trials performed by van Dijken and Hickel et al (20,21). On the other hand, Soares et al found that MOD cavities restored with resin composite placed with direct technique attained better biomechanical performance than those restored with laboratory processed resin and ceramic restorations (15,16). Another study also confirmed that in comparison with ceramic restorations, resin composite restoration had higher fatigue resistance (17). As opposed to preparation for direct restoration, cavity preparation for indirect restorations requires removal of additional amount of tooth structure (22). The situation is the same with the cavity preparation for the amalgam (23). Since the quantity of the tooth structure removed while doing cavity preparation affects the biomechanical characteristics of the restored tooth, the use of adhesive direct restorations should be recommended for reinforcing the remaining dental structure (2,16).

When planning the design of MOD cavity preparation, cavity wall thickness and cusp reduction should be carefully considered. Usually, cusp reduction is recommended when cavity isthmus width is 2/3 of intercuspal width (7,24). Although this promotes more dental tissue reduction (25), it was shown that the reduction of cuspal height by 2.0 mm increases fracture resistance of a premolar when restored with direct resin composite (26,27). On the other hand, cavity wall thickness is not well defined. Macpherson et al found that 2.25 mm wall thickness is critical for restoring fracture resistance of tooth with MOD cavity (28), but another study, which investigated 1.0-3.0 mm wall thicknesses, reported that the thickness of remaining cavity walls was not relevant to fracture resistance (27).

The aim of this study was to investigate the effect of cavity design preparation on stress values in remaining tooth structures restored with resin composite. The null hypothesis was that stress values were not significantly influenced by the cavity wall thickness and cusp reduction.

## Material and methods

The study was conducted at the Clinic of Dentistry of Vojvodina, Novi Sad, Serbia, and at the Institute of Physics, Belgrade, Serbia, from December 2011 to March 2012. A human maxillary second premolar, extracted for orthodontic reasons, at the Clinic of Dentistry of Vojvodina was used for the study. Immediately after the extraction, the tooth was cleaned of soft tissue remnants and used for 3D solid model generation. The selected tooth was intact, without caries, fractures, and morphological abnormalities.

### 3D solid model generation

The extracted tooth was scanned using multilayer CT scanner (SOMATOM Sensation 64 Cardiac, Siemens, Forchheim, Germany). A total of 110 slices were made along x-axis, 88 along y-axis, and 47 along z axis. For the solid model generation, slices along z axis were used with 0.5 mm resolution. The selected slices were imported to AMIRA software (Visage Imaging Inc, San Diego, CA, USA) for automatic tooth structures (enamel, dentin, pulp) segmentation. The segmentation was based on image density threshold of different gray scale intensities corresponding to various degrees of mineralization. Obtained contours were then imported into SolidWorks 2011 software (Dassault Systems SolidWorks Corp, Waltham, MA, USA), and 3D solid model of the intact maxillary second premolar was generated by using a lofting technique. Additionally, based on the outer geometry of the model, periodontal ligament and alveolar bone (cortical and cancellous) were created.

### Cavity preparation design

**Figure 1:**
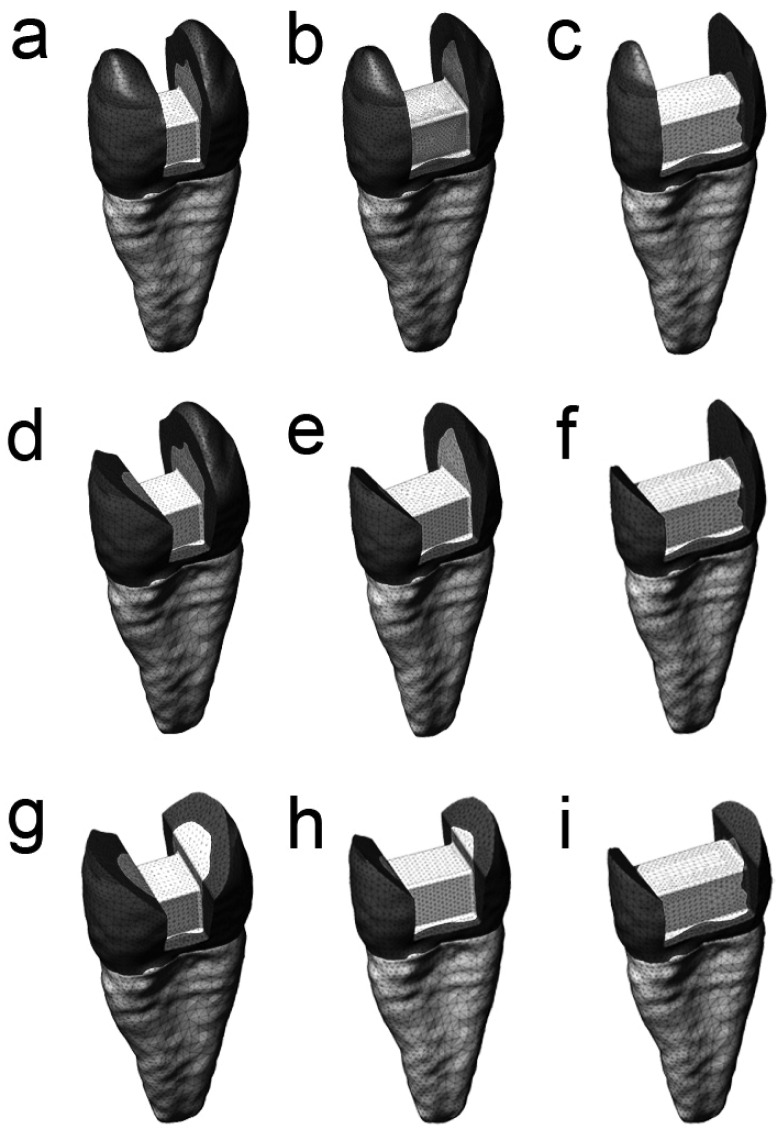
**3D solid tooth models with different mesio-occlusal-distal (MOD) cavities. (A**) Model 1: 3D solid tooth model with MOD cavity with 3.0 mm wall thickness, without cusp reduction; (**B**) Model 2: 3D solid tooth model with MOD cavity with 2.25 mm wall thickness, without cusp reduction; (**C**) Model 3: 3D solid tooth model with MOD cavity with 1.5 mm wall thickness, without cusp reduction; (**D**) Model 4: 3D solid tooth model with MOD cavity with 3.0 mm wall thickness and 2.0 mm palatal cusp reduction; (**E**) Model 5: 3D solid tooth model with MOD cavity with 2.25 mm wall thickness and 2.0 mm palatal cusp reduction; (**F**) Model 6: 3D solid tooth model with MOD cavity with 1.5 mm wall thickness and 2.0 mm palatal cusp reduction; (**G**) Model 7: 3D solid tooth model with MOD cavity with 3.0 mm wall thickness and 2.0 mm palatal and buccal cusp reduction; (**H**) Model 8: 3D solid tooth model with MOD cavity with 2.25 mm wall thickness and 2.0 mm palatal and buccal cusp reduction; (**I**) Model 9: 3D solid tooth model with MOD cavity with 1.5 mm wall thickness and 2.0 mm palatal and buccal cusp reduction.

In the 3D solid model of intact maxillary premolar, different MOD cavities were designed. All MOD cavities had pulpal and axial walls with at least 1.0 mm dentin thickness over the pulp (29), while the gingival walls were located 1.0 mm above cemento-enamel junction (CEJ). Solid models of MOD cavities were created with three different wall thicknesses (3.0 mm, 2.25 mm, and 1.5 mm), and three different cusp reduction procedures (without cusp reduction, 2.0 mm palatal cusp reduction, and 2.0 mm palatal and buccal cusp reduction). In total, there were nine 3D solid tooth models (Figure 1). Restorations of all MOD cavities were simulated as direct resin composite restorations (Gradia Direct Posterior, GC, Tokyo, Japan).

### Finite element analysis

Nine 3D solid tooth models with MOD cavities restored with resin composite were used to simulate nine clinically different cavity designs. All 3D solid models were derived from the 3D solid model of the intact maxillary second premolar. 3D solid models were meshed with parabolic tetrahedral elements. The parabolic tetrahedral element is defined by four corner nodes, six mid-side nodes, and six edges. These elements were used because they represent curved boundaries more accurately and provide better mathematical approximations. The number of elements and nodes varied according to the model (142 407-175 727 elements and 223 113-268 918 nodes). Convergence test was used to verify that our numerical model reached converged results and that no further mesh refinement was necessary. The exterior nodes on all surfaces of the cortical bone were restrained in all directions as the boundary conditions for all models. All materials were assumed to have linear, elastic, and isotropic properties (7,8,10), represented by the Young's modulus of elasticity and the Poisson's ratio (Table 1) (7,14,30).

**Table 1:** Material properties assigned to dental tissues and restorative material

Material	Young's modulus (MPa)	Poisson's ratio	References
Enamel	84,100	0.20	(7,14)
Dentin	18,600	0.31	(7,14)
Pulp	2	0.45	(7,14)
Periodontal ligament	70	0.45	(7,14)
Cortical bone	15,000	0.30	(7,14)
Cancellous bone	1500	0.30	(7,14)
Resin composite	6700	0.22	(30)

To simulate masticatory forces in the maximum intercuspation position, the static axial load with the resulting force of 200 N was applied on the occlusal surface of a tooth at three points (palatal cusp tip and both marginal ridges) (31,32). Using structural FEA, maximum von Misses stress values in the enamel, dentin, and resin composite were calculated.

### Statistical methods

To investigate the impact of cavity design parameters on stress values in maxillary premolar, a full three-level factorial design based on a quadratic model was used with a total of nine (3^2^) experiments (33). Two design factors were considered: cavity wall thickness and cuspal reduction. Each design factor was assigned three levels (Table 2).

**Table 2:** Factorial design – investigated factors and assigned levels

Investigated factor		Level		
	1	2	3	
Cavity wall thickness (mm)	1.50	2.25	3.00	
Cuspal reduction (mm)	None	Palatal (2.00)	Palatal and buccal (2.00)	

In order to establish the relative importance of the investigated factors and their interactions, analysis of variance (ANOVA) was conducted for enamel, dentin, and resin composite stress values, respectively (7). Factorial analysis was conducted in Statistica v10 software (Statsoft Inc, Tulsa, OK, USA).

## Results

The von Mises stresses in enamel, dentin, and resin composite, obtained by FEA, were 79.3-233.6 MPa, 26.0-32.9 MPa, and 180.2-252.2 MPa, respectively (Table 3).

**Table 3:** Factorial experiments in randomized order, with respective output stresses obtained by finite element analysis

Run	Cavity wall thickness (mm)	Cuspal reduction	Enamel stress (MPa)	Dentin stress (MPa)	Resin composite stress (MPa)
1	2.25	palatal	79.3	26.0	180.8
2	1.50	palatal and buccal	83.4	32.8	199.7
3	3.00	palatal and buccal	82.7	27.2	196.6
4	2.25	none	196.6	32.9	188.9
5	2.25	palatal and buccal	87.8	26.9	182.5
6	1.50	none	233.6	32.1	252.2
7	1.50	palatal	94.2	28.7	180.2
8	3.00	palatal	83.2	27.5	195.2
9	3.00	none	172.5	27.7	210.3

**Figure 2:**
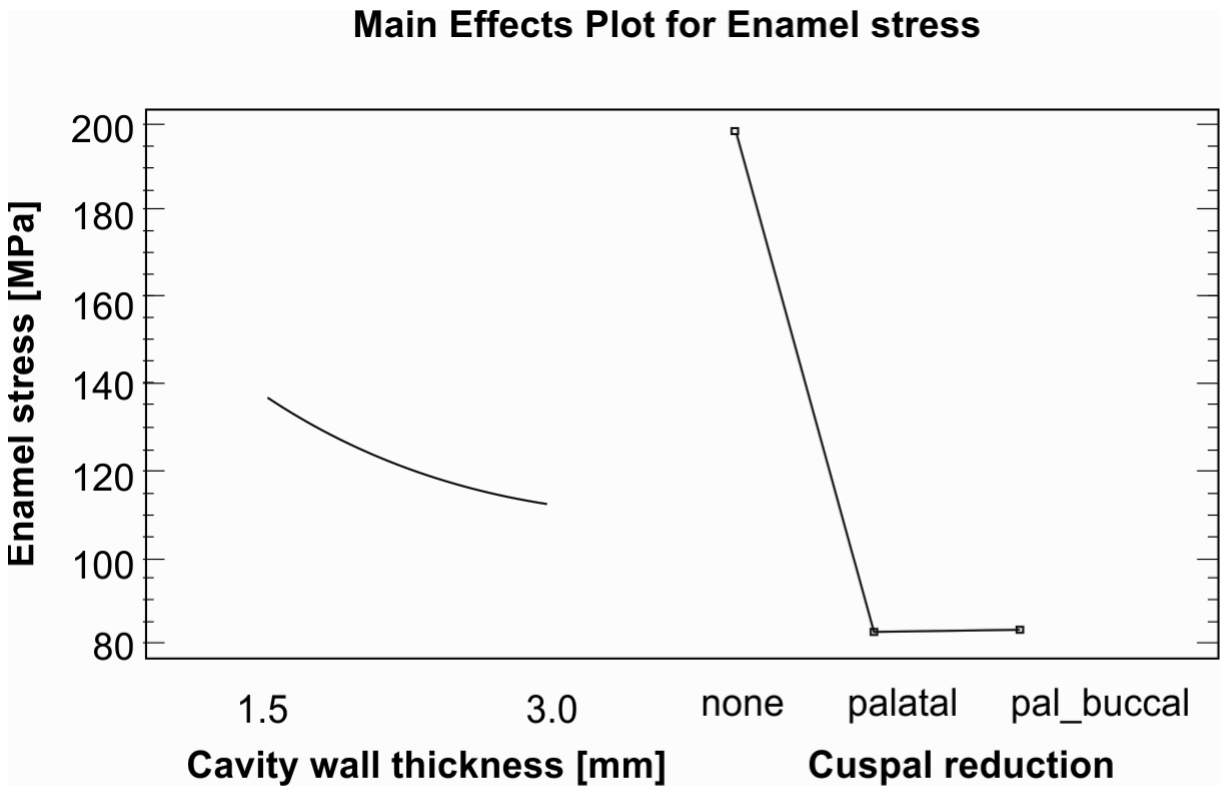
Main effects plot for enamel stress.

**Figure 3:**
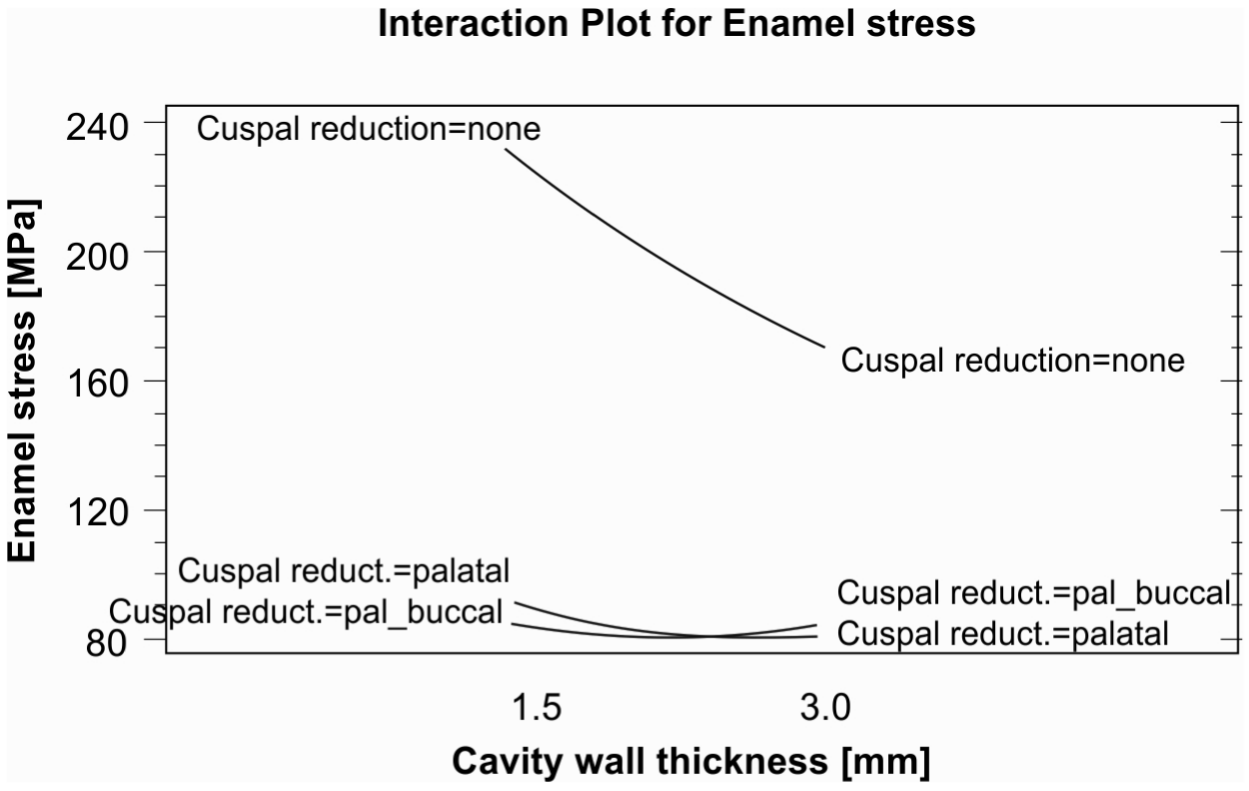
Interaction plot for enamel stress.

Considering the influence of cavity design parameters on enamel stress, the ANOVA showed that cuspal reduction (92.97%) and cavity wall thickness (3.06%) significantly (*P* < 0.05) determined the magnitude of stress values (Table 4). The main effects plot for enamel stress (Figure 2) revealed that the increased cavity wall thickness moderately reduced enamel stress, while the presence of palatal or palatal and buccal cusp reduction contributed to lower stress values, compared to the case with no cuspal reduction. Interaction plot (Figure 3) showed that at maximum wall thickness value, application of palatal and buccal cusp reduction resulted in minimal stress values.

**Table 4:** ANOVA statistical results of von Mises stresses for enamel

Source	Degrees of freedom	Sum of squares	Mean sum of squares	Total sum of squares (%)	*P*
Cavity wall thickness	1	883.31	883.31	3.06	0.0396
Cuspal reduction	2	26820.60	13410.30	92.97	0.0028
Cavity wall thickness × cavity wall thickness	1	27.38	27.38	0.10	0.4811
Cavity wall thickness × cuspal reduction	2	1044.04	522.02	3.61	0.0664
Residual	2	74.30	37.15	0.26	
Total	8	28849.63		100.00	

**Figure 4:**
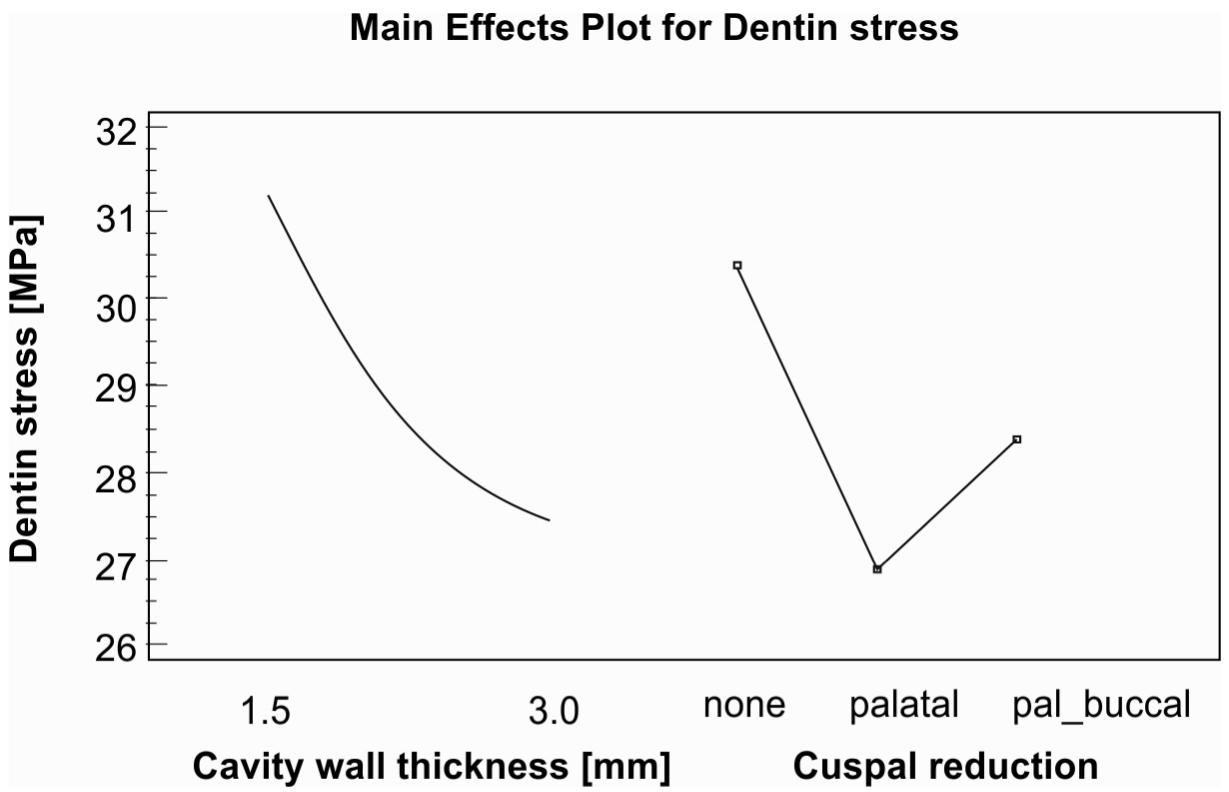
Main effects plot for dentin stress.

**Figure 5:**
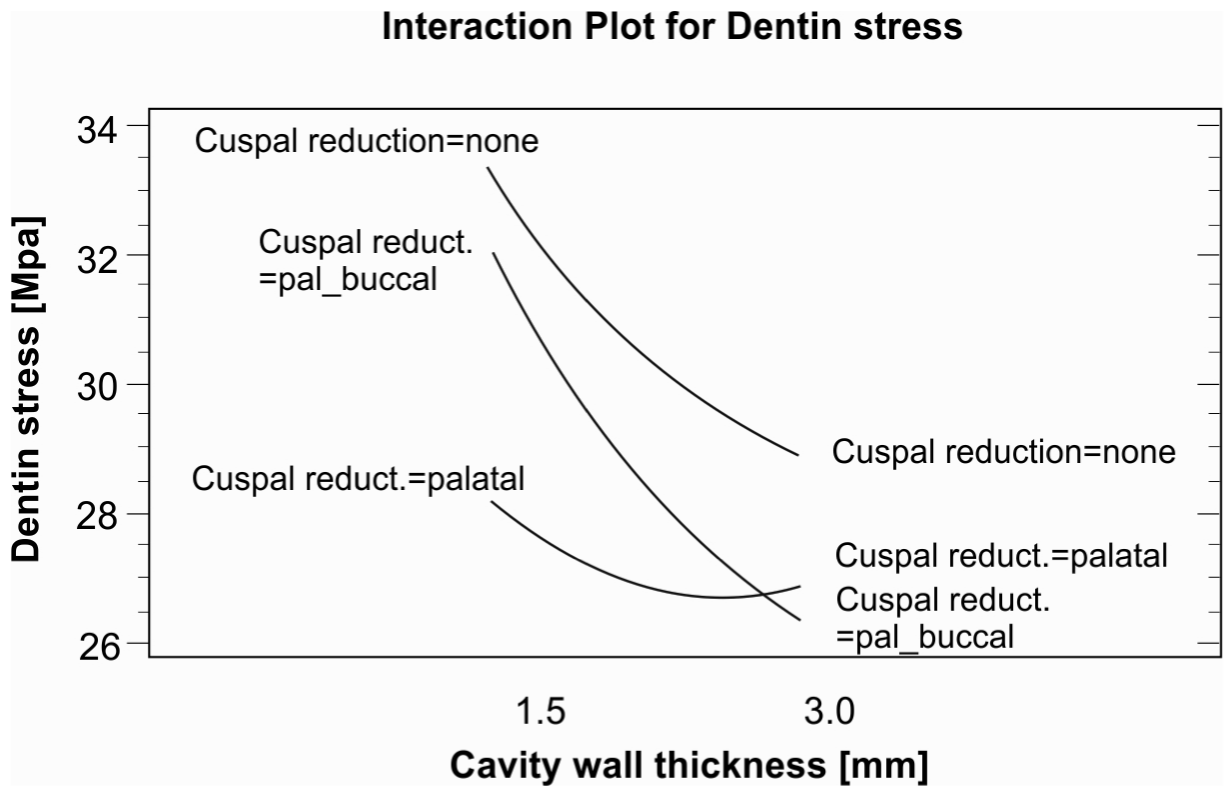
Interaction plot for dentin stress.

For the stress values of dentin, the results also showed that cavity wall thickness (34.92%) and cuspal reduction (30.80%) affected the magnitude of stress values (Table 5). However, no significant association (*P* < 0.05) was established. The main effects plot for dentin stress again revealed that the increase of cavity wall thickness resulted in lower stresses, while in the case of cuspal reduction, palatal cusp reduction resulted in lower stress values (Figure 4). The interaction plot (Figure 5) indicated that, at maximum cavity wall thickness, the application of palatal and buccal cusp reduction resulted in the lowest stress values.

**Table 5:** ANOVA statistical results of von Mises stresses for dentin

Source	Degrees of freedom	Sum of squares	Mean sum of squares	Total sum of squares (%)	*P*
Cavity wall thickness	1	20.91	20.91	34.92	0.2291
Cuspal reduction	2	18.44	9.22	30.80	0.4362
Cavity wall thickness × cavity wall thickness	1	1.08	1.07	1.80	0.7353
Cavity wall thickness × cuspal reduction	2	5.17	2.59	8.64	0.7339
Residual	2	14.27	7.14	23.84	
Total	8	59.87		100.00	

**Figure 6:**
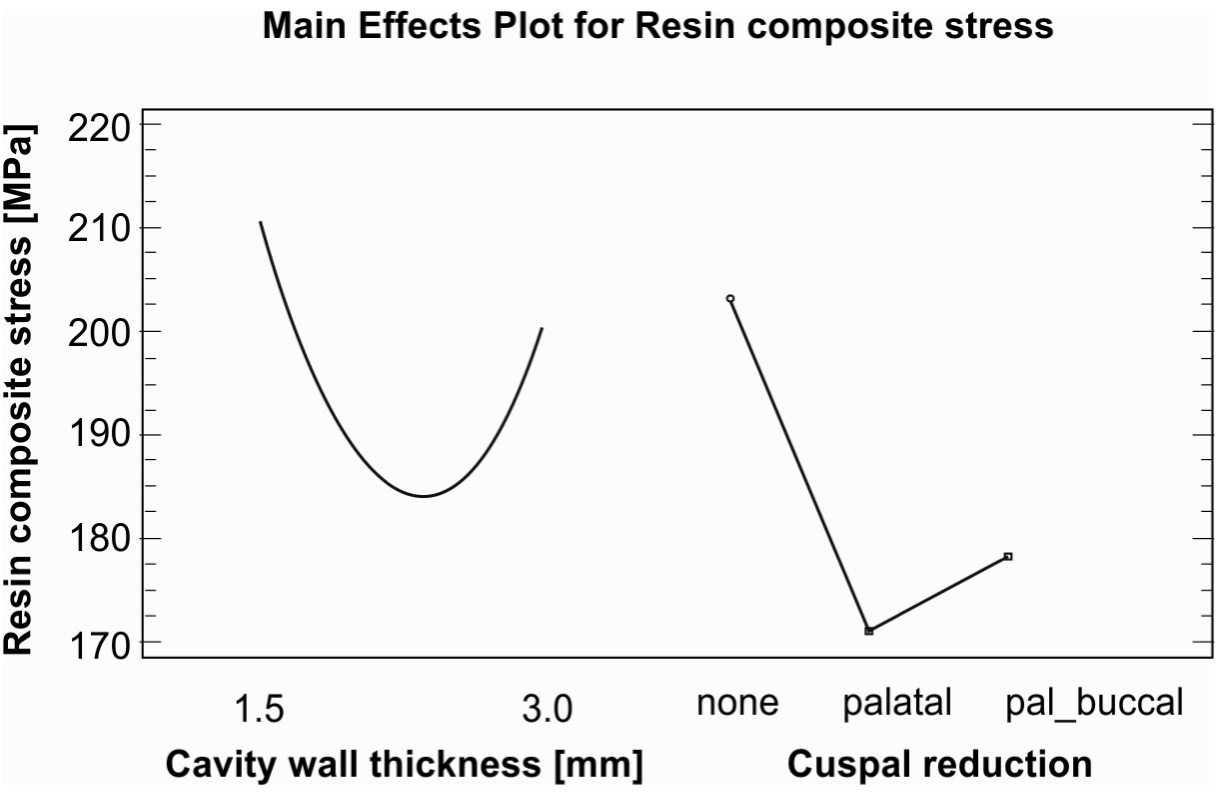
Main effects plot for resin composite stress.

**Figure 7:**
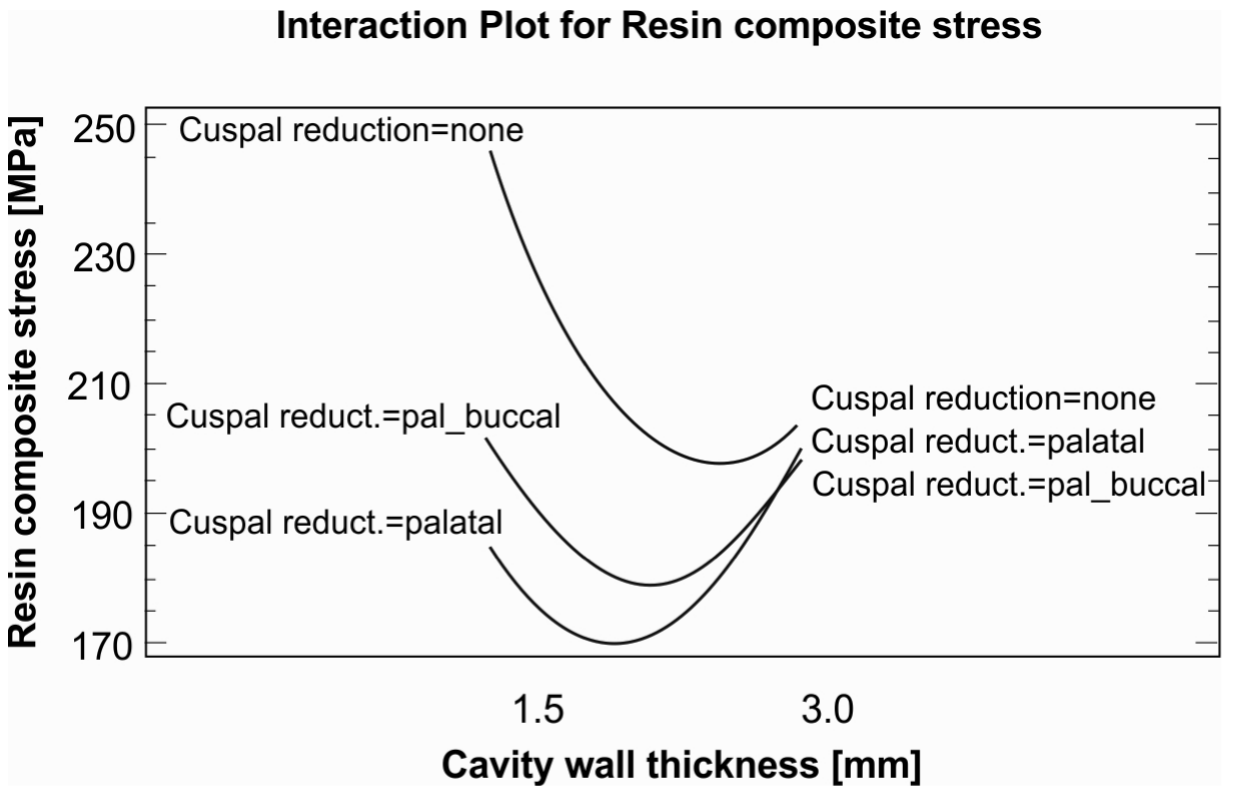
Interaction plot for resin composite stress.

Regarding the influence of cavity design on restorative material, the analysis of stress values in resin composite revealed the dominant effect of cuspal reduction (40.87%), followed by interaction of cavity wall thickness and cuspal reduction (20.94%), and cavity wall thickness (3.72%) (Table 6). However, as in the case of dentin stress, no significant association (*P* < 0.05) was established. The main effects plot showed that the minimum stress was obtained for medium cavity wall thickness, ie, the palatal cusp reduction (Figure 6). From the interaction plot (Figure 7) it is also evident that at medium cavity wall thickness, the most favorable stress was obtained for palatal cusp reduction.

**Table 6:** **ANOVA statistical results of von Mises stresses for resin composite**

Source	Degrees of freedom	Sum of squares	Mean sum of squares	Total sum of squares (%)	*P*
Cavity wall thickness	1	150.00	150.00	3.72	0.5019
Cuspal reduction	2	1649.40	824.70	40.87	0.2161
Cavity wall thickness × cavity wall thickness	1	936.00	936.00	23.20	0.1796
Cavity wall thickness × cuspal reduction	2	845.11	422.56	20.94	0.3498
Residual	2	454.70		11.27	
Total	8	4035.21		100.00	

**Figure 8:**
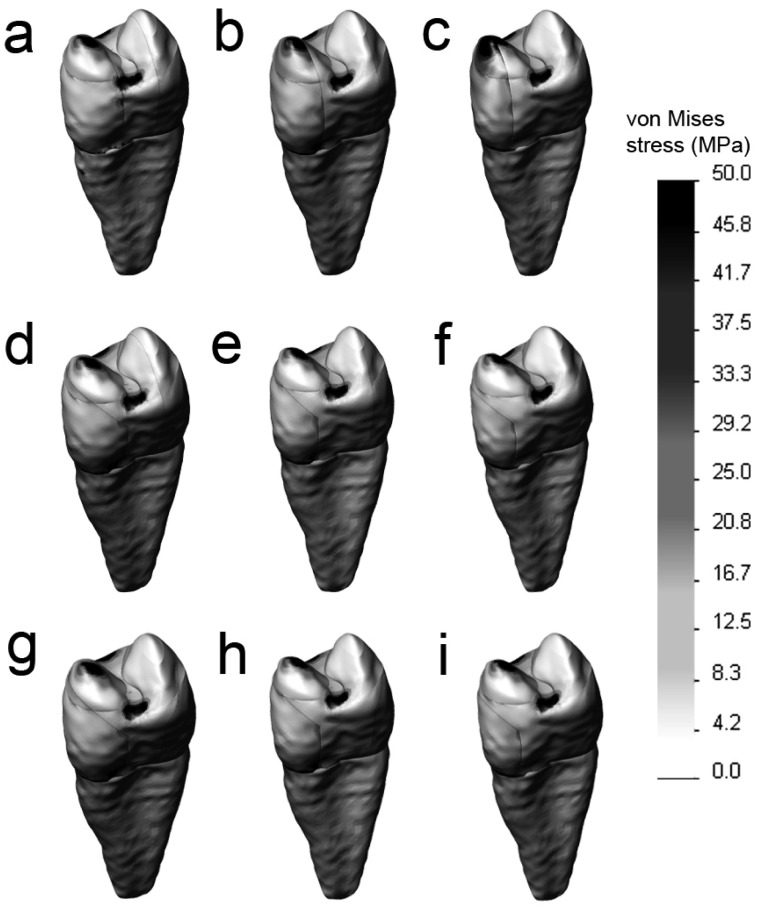
**Von Mises stress distribution results** (**A**) Model 1: 3D solid tooth model with mesio-occlusal-distal (MOD) cavity with 3.0 mm wall thickness, without cusp reduction; (**B**) Model 2: 3D solid tooth model with MOD cavity with 2.25 mm wall thickness, without cusp reduction; (**C**) Model 3: 3D solid tooth model with MOD cavity with 1.5 mm wall thickness, without cusp reduction; (**D**) Model 4: 3D solid tooth model with MOD cavity with 3.0 mm wall thickness and 2.0 mm palatal cusp reduction; (**E**) Model 5: 3D solid tooth model with MOD cavity with 2.25 mm wall thickness and 2.0 mm palatal cusp reduction; (**F**) Model 6: 3D solid tooth model with MOD cavity with 1.5 mm wall thickness and 2.0 mm palatal cusp reduction; (**G**) Model 7: 3D solid tooth model with MOD cavity with 3.0 mm wall thickness and 2.0 mm palatal and buccal cusp reduction; (**H**) Model 8: 3D solid tooth model with MOD cavity with 2.25 mm wall thickness and 2.0 mm palatal and buccal cusp reduction; (**I**) Model 9: 3D solid tooth model with MOD cavity with 1.5 mm wall thickness and 2.0 mm palatal and buccal cusp reduction.

Stress distribution patterns were similar for all models (Figure 8). The highest stress values occurred at loading points (palatal cusp tip and both marginal ridges) and at cervical area of the palatal surface.

## Discussion

The present study indicated that cavity design preparation generally affected von Mises stress values in premolar restored with direct resin composite. While the cusp reduction decreased stress values and provided more favorable stress distribution, cavity wall thickness showed no significant influence.

Preservation of sound tooth structures is the primary goal of modern restorative dentistry. However, from biomechanical point of view, protection of remaining tooth structures from unfavorable mechanical responses should be considered a priority, even if it requires the removal of additional dental tissue (7,17). Due to their unfavorable anatomy, maxillary premolars with extensive MOD cavities are at great risk of fracturing if restored without regarding protective principles (1,22). It is well known that palatal cusp of maxillary premolars fractures more frequently than buccal cusp (7,34). Also, Soares et al experimentally confirmed that palatal cusp was involved in more severe type of fracture than buccal cusp (15). In order to prevent this, several studies recommended cusp reduction from at least 1.5-3.5 mm (7,17,26,27). In this way, higher fracture resistance of restored tooth can be accomplished (17,26,27). Even if fracture occurs, it is going to be less severe than without cusp reduction. Some studies support the coverage of palatal cusp only (1,27,35), while others assert coverage of both cusps (17,26).

In the present study, the cusp reduction in 3D solid models decreased stress values, but significant relationship was found only for enamel. The obtained stress values were much lower than those calculated for the enamel of intact tooth model (155 MPa). Although these results indicated similar effect of palatal, and both palatal and buccal cusp height reduction, when von Mises stresses for the enamel, dentine, and resin composite were considered in conjunction, palatal cusp coverage was proven to be the optimal option. Stress distribution patterns confirmed this finding. Although location of highest stress values was similar for all models, stress concentration areas at palatal cusp tip for models without cusp reduction were wider than for the models with cusp coverage, especially if tooth-restoration interface was in contact with applied force. Further, in models with cusp reduction, stress concentration areas were relocated from the remaining tooth structures to the restoration. This should preserve remaining tooth structures from unfavorable mechanical responses, which could make tooth non-restorable (7). Also, no differences were found in stress distribution at buccal cusp. This, again, implies that only palatal cusp should be reduced in wide MOD cavities in maxillary premolars.

Regarding the cavity wall thickness, the results showed that this parameter of cavity design was not as relevant as the cusp reduction, which is in accordance with the findings of other authors (14,27).

In the present study, direct resin composite restoration was simulated for all types of cavity preparations. The use of this material provides 83.3% fracture resistance of sound tooth (15), and stress distribution similar to that of a sound tooth (2,16). Further, cavity preparation for direct resin composite restoration requires less tooth structure removal than for the amalgam or indirect ceramic restoration, which is in accordance with the main principle of modern restorative dentistry. Also, resin composites have good clinical survival rate (90% after two years and 55.1%-89.7% after ten years), even when they are used for restoration of extensive cavities in posterior teeth (36,37).

The 3D solid tooth model was generated using CT scan-based data. It presents a modern approach to achieving a highly detailed 3D finite element model of a tooth (5,10,38), and has an important role in investigations of different clinical situations in dentistry (8,12). Numerical modeling and simulation is useful for obtaining information about mechanical behavior of sound and restored tooth (39), and is able to demonstrate the otherwise inaccessible stress distribution within the tooth-restoration complex (11). It also saves time and costs related to experimental studies and clinical trials (5). Furthermore, this method allows an infinite number of variables to be studied (6). On the other hand, it is obviously quite impossible to include all of the factors from the oral environment in a computer simulation (40). Moreover, it is known that factors such as material properties and loading conditions significantly influence the FEA results (14,40). Thus, numerical prototyping seems to be a valid method for bringing a new idea from concept to clinical application (11).

In conclusion, based on the numerical simulations and analysis applied in this study, a palatal cusp reduction could be suggested in order to reveal a lower stress values in dental tissues and restorative material. With the respect to the sound tooth structures, this type of cavity design should contribute to better biomechanical behavior of a tooth-restoration complex, consequently providing the long-lasting clinical results.

## References

[1] Bitter K, Meyer-Lueckel H, Fotiadis N, Blunck U, Neumann K, Kielbassa AM (2010). Influence of endodontic treatment, post insertion, and ceramic restoration on the fracture resistance of maxillary premolars.. Int Endod J.

[2] Soares PV, Santos-Filho PC, Queiroz EC, Araujo TC, Campos RE, Araujo CA (2008). Fracture resistance and stress distribution in endodontically treated maxillary premolars restored with composite resin.. J Prosthodont.

[3] Jiang W, Bo H (2010). YongChun G, LongXing N. Stress distribution in molars restored with inlays or onlays with or without endodontic treatment: A three-dimensional finite element analysis.. J Prosthet Dent.

[4] Magne P (2007). Efficient 3D finite element analysis of dental restorative procedures using micro-CT data.. Dent Mater.

[5] Ausiello P, Franciosa P, Martorelli M, Watts DC (2011). Numerical fatigue 3D-FE modeling of indirect composite-restored posterior teeth.. Dent Mater.

[6] Magne P, Oganesyan T (2009). CT scan-based finite element analysis of premolar cuspal deflection following operative procedures.. Int J Periodontics Restorative Dent..

[7] Lin CL, Chang YH, Liu PR (2008). Multi-factorial analysis of a cusp-replacing adhesive premolar restoration: A finite element study.. J Dent.

[8] Ausiello P, Apicella A, Davidson CL (2002). Effect of adhesive layer properties on stress distribution in composite restorations-a 3D finite element analysis.. Dent Mater.

[9] Dejak B, Mlotkowski A (2008). Three-dimensional finite element analysis of strength and adhesion of composite resin versus ceramic inlays in molars.. J Prosthet Dent.

[10] Rodrigues FP, Li J, Silikas N, Ballester RY, Watts DC (2009). Sequential software processing of micro-XCT dental-images for 3D-FE analysis.. Dent Mater.

[11] Magne P (2010). Virtual prototyping of adhesively restored, endodontically treated molars.. J Prosthet Dent.

[12] Kantardzic I, Vasiljević D, Blazic L, Puskar T, Tasic M (2012). Computed-tomography scan-based finite element analysis of stress distribution in premolars restored with composite resin.. Physica Scripta.

[13] Wakabayashi N, Ona M, Suzuki T, Igarashi Y (2008). Nonlinear finite element analysis: advances and challenges in dental applications.. J Dent.

[14] Lin CL, Chang WJ, Lin YS, Chang YH, Lin YF (2009). Evaluation of the relative contributions of multi-factors in an adhesive MOD restoration using FEA and the Taguchi method.. Dent Mater.

[15] Soares PV, Santos-Filho PC, Martins LR, Soares CJ (2008). Influence of restorative technique on the biomechanical behavior of endodontically treated maxillary premolars. Part I: fracture resistance and fracture mode.. J Prosthet Dent.

[16] Soares PV, Santos-Filho PC, Gomide HA, Araujo CA, Martins LR, Soares CJ (2008). Influence of restorative technique on the biomechanical behavior of endodontically treated maxillary premolars. Part II: strain measurement and stress distribution.. J Prosthet Dent.

[17] Magne P, Knezevic A (2009). Thickness of CAD-CAM composite resin overlays influences fatigue resistance of endodontically treated premolars.. Dent Mater.

[18] Cubas GB, Habekost L, Camacho GB, Pereira-Cenci T (2011). Fracture resistance of premolars restored with inlay and onlay ceramic restorations and luted with two different agents.. J Prosthodont Res..

[19] Kuijs RH, Fennis WM, Kreulen CM, Roeters FJ, Verdonschot N, Creugers NH (2006). A comparison of fatigue resistance of three materials for cusp-replacing adhesive restorations.. J Dent.

[20] van Dijken JW (2000). Direct resin composite inlays/onlays: an 11 year follow-up.. J Dent.

[21] Hickel R, Manhart J (2001). Longevity of restorations in posterior teeth and reasons for failure.. J Adhes Dent.

[22] Mondelli J, Sene F, Ramos RP, Benetti AR (2007). Tooth structure and fracture strength of cavities.. Braz Dent J.

[23] Lynch CD, Frazier KB, McConnell RJ, Blum IR, Wilson NH (2010). State-of-the-art techniques in operative dentistry: contemporary teaching of posterior composites in UK and Irish dental schools.. Br Dent J.

[24] Roberson TM, Heymann HO, Ritter AV, Pereira PN. Class I, II, and VI direct composite and other tooth-colored restorations. In: Roberson TM, Heymann HO, Swift EJ, editors. Sturdevant's art and science of operative dentistry. Maryland Heights (MO): Mosby Inc; 2006. p 594.

[25] Seow LL, Toh CG, Wilson NH (2005). Remaining tooth structure associated with various preparation designs for the endodontically treated maxillary second premolar.. Eur J Prosthodont Restor Dent.

[26] Mondelli RF, Ishikiriama SK, de Oliveira Filho O, Mondelli J (2009). Fracture resistance of weakened teeth restored with condesable resin with and without cusp coverage.. J Appl Oral Sci.

[27] ElAyouti A, Serry MI, Geis-Gerstorfer J, Lost C (2011). Influence of cusp coverage on the fracture resistance of premolars with endodontic access cavities.. Int Endod J.

[28] Macpherson LC, Smith BG (1995). Reinforcement of weakened cusps by adhesive restorative materials: an in-vitro study.. Br Dent J.

[29] Murray PE, Smith AJ (2002). Saving pulps-a biological basis. An overview.. Prim Dent Care.

[30] Sorrentino R, Aversa R, Ferro V, Auriemma T, Zarone F, Ferrari M (2007). Three-dimensional finite element analysis of strain and stress distributions in endodontically treated maxillary central incisors restored with different post, core and crown materials.. Dent Mater.

[31] Myers GE, Anderson JR (1971). Nature of contacts in centric occlusion in 32 adults.. J Dent Res.

[32] Sturdevant JR. Clinical significance of dental anatomy, histology, physiology, and occlusion. In: Roberson TM, Heymann HO, Swift EJ, editors. Sturdevant's art and science of operative dentistry. Maryland Heights (MO): Mosby Inc; 2006.p 42.

[33] Dar FH, Meakin JR, Aspden RM (2002). Statistical methods in finite element analysis.. J Biomech.

[34] Deliperi S, Bardwell D, Coiana C (2005). Reconstruction of devital teeth using direct fiber-reinforced composite resins: A case report.. J Adhes Dent.

[35] Fennis WM, Kuijs RH, Kreulen CM, Verdonschot N, Creugers NH (2004). Fatigue resistance of teeth restored with cuspal-coverage composite restorations.. Int J Prosthodont.

[36] Kubo S (2011). Longevity of resin composite restorations.. Jpn Dent Sci Rev.

[37] Nagasiri R, Chitmongkolsuk S (2005). Long-term survival of endodontically treated molars without crown coverage: A retrospective cohort study.. J Prosthet Dent.

[38] Poiate IA, Vasconcellos AB, Mori M, Poiate E (2011). 2D and 3D finite element analysis of central incisor generated by computerized tomography.. Comput Methods Programs Biomed.

[39] Ausiello P, Apicella A, Davidson CL, Rengo S (2001). 3D-finite element analysis of cusp movements in a human upper premolar, restored with adhesive resin-based composites.. J Biomech.

[40] Dejak B, Mlotkowski A, Langot C (2012). Three-dimensional finite element analysis of molars with thin-walled prosthetic crowns made of various materials.. Dent Mater.

